# Influence of Functional Classification on Skill Tests in Elite Female Wheelchair Basketball Athletes

**DOI:** 10.3390/medicina55110740

**Published:** 2019-11-15

**Authors:** Kaori Tachibana, Hirotaka Mutsuzaki, Yukiyo Shimizu, Takashi Doi, Kazushi Hotta, Yasuyoshi Wadano

**Affiliations:** 1 Department of Physical Therapy, School of Healthcare, Ibaraki Prefectural University of Health Sciences, Ami 300-0394, Japan; 2 Center for Medical Sciences, Ibaraki Prefectural University of Health Sciences, Ami 300-0394, Japan; 3 Department of Orthopedic Surgery, Ibaraki Prefectural University of Health Sciences Hospital, Ami 300-0331, Japan; 4 Department of Rehabilitation Medicine, University of Tsukuba Hospital, Tsukuba 305-8577, Japan; 5 Department of Occupational Therapy, School of Healthcare, Ibaraki Prefectural University of Health Sciences, Ami 300-0394, Japan; 6 Department of Orthopedic Surgery, Miho Clinic, Miho 300-0415, Japan

**Keywords:** wheelchair basketball, functional classification system, performance, field-based tests, functional ability

## Abstract

*Background and objectives:* Wheelchair basketball players are classified into four functional classes according to the player’s “volume of action”, characterized by trunk movement and stability. As this classification is based on a kinematic point of view, test items might be differentially affected by the functional classification level. We aimed to clarify field-based skill test items closely related to the functional classification level. *Materials and Methods:* Twenty-six female wheelchair basketball athletes (Japan National Team candidates) completed seven field-based skill tests (20 m sprint, agility T-test, figure-eight with a ball test, the Yo-Yo 10 m recovery test, and three types of maximal passes), and anthropometric measurements were applied. *Results:* Significant differences among the classification levels were found for one-hand maximal passes (baseball and hook passes) and the figure-eight with a ball test. Furthermore, performance in the 20 m sprint and 10 m Yo-Yo recovery tests significantly differed between classes 1 and 4. *Conclusions:* The test items most influenced by the classification levels were one-hand passes, which require trunk stability and balance not only in the horizontal plane, but also in the sagittal and frontal planes. Coaches should consider which test items are strongly affected by the functional classification level when assessing a player’s performance.

## 1. Introduction

Wheelchair basketball athletes with mixed characteristics are divided into five main classes (1.0, 2.0, 3.0, 4.0, and 4.5, with 4.5 reflecting the greatest level of functional ability) and three subclasses (1.5, 2.5, and 3.5), in accordance with the International Wheelchair Basketball Federation (IWBF) official player classification manual [1]. Trunk, lower limb, and upper limb function determine a player’s class; however, trunk movement and stability are particularly important for determining the classification. By definition, a class 1.0 player has little voluntary control in any plane and, therefore, relies on his/her wheelchair and/or his/her arms for support in all planes of movement. A class 2.0 player has active upper trunk rotation, which allows for a partial volume of action in the transverse and sagittal planes. A class 3.0 player has a full volume of action in the transverse and sagittal planes, but no volume of action in the frontal plane. A class 4.0 player has a complete volume of action in the transverse and sagittal planes and a full volume of action to one side, while a class 4.5 player has a complete volume of action in all planes. In wheelchair basketball, each player’s functional class was evaluated by the practice and game observation during competition. In order to achieve fair and objective classification, it is important that the classifier knows the movement characteristics of each class of player and what movements are affected by the class difference.

The validity of the wheelchair basketball functional classification system has been considered from various points of view, e.g., physiology [2,3,4,5], biomechanics [6,7], and game performance [8,9,10]. Several studies on wheelchair basketball athletes have shown that the classification level and anaerobic performance are correlated to some extent [3,4]. Vanlandewijck et al. [10] investigated whether the functional classification reflects the differences in the performance of elite female wheelchair basketball players in the World Championships, Sydney, 1988. The authors demonstrated that high-point players perform better than low-point players for the majority of variables that determine the quality of game performance in elite female wheelchair basketball players.

Other studies have examined the differences among classes using field-based skill tests that are specific to wheelchair basketball [11,12,13,14,15]. Most studies have reported a strong relationship between field-based testing and functional class; however, the participants were divided into only two class categories (low-point player: classes 1.0–2.5; high-point player: classes 3.0–4.5) [12,15,16]. Thus, the relationship between the basic IWBF functional classification level and performance on each field-based test item remains unclear. Additionally, Gil et al. [13] found that the functional classification level was correlated to performance on grip force, maximal pass, and medicine ball throw test items, while sprint and agility test items were correlated to the years of wheelchair dependence or experience in wheelchair basketball. Cavadon et al. [14] examined Italy’s young players to evaluate the anthropometry, body composition, and field test performance with functional classification and game-related statistics. They insisted that sitting height and functional ability especially correlated with performance outcomes; however, there was a large overlap in both sport-specific field test performances in middle to high functional class. Several of these previous studies [3,12,14,15] have a cross-sectional nature, examining a single point in time. Thus, performance in field-based skill tests may include the effects of inexperience and aging, and it remains unclear which test items are strongly influenced by a player’s functional ability. A better understanding of this issue would aid coaches and classifiers in interpreting test results as strongly representing the athlete’s functional classification level or their effort.

Thus, this study aimed to clarify the field-based skill test items closely related to the functional classification level in elite female wheelchair basketball players. We evaluated four years of field-based skill test performance of elite female athletes according to the IWBF basic functional classes.

## 2. Materials and Methods

### 2.1. Participants

Between 2015 and 2018, a total of 37 female wheelchair basketball athletes from the local women’s wheelchair basketball teams in Japan were recommended by their local coaches to participate in the national team selection camp, which is held once a year (usually in November or December). During the camp, the athletes were evaluated by the national team coaching staff using field-based skill tests and a game performance. Demographic data of the participants were obtained from medical check-ups, which were conducted by the national team doctor prior to each selection camp. As a result of the selection process, 27 athletes were selected as national team candidates and participated in the national team training camps at least once in the four-year study period. In the assessment of data normality, an extreme deviation from the mean was observed for class 3.5 athletes. Thus, these data were excluded, and further statistical examination was performed using data from the 26 athletes.

For this study, the players were grouped into four functional classes: Class 1 (*n* = 7) comprised of classes 1.0 and 1.5, class 2 (*n* = 7) included classes 2.0 and 2.5, class 3 (*n* = 5) contained classes 3.0 and 3.5, and class 4 (*n* = 8) consisted of classes 4.0 and 4.5.

The study protocol was approved by the Human Ethics Review Committee of Ibaraki Prefectural University of Health Sciences (Approval Nos. 485 from 13^th^ April 2014 and e45 from 30^th^ of May 2016) and was conducted according to the principles expressed in the Declaration of Helsinki. All participants provided written informed consent.

### 2.2. Field-Based Skill Tests

On the first day of camp, participants completed a series of field-based skill tests to evaluate their speed, agility, endurance, and long-throw ability. All participants used their own basketball wheelchairs. Before each testing session, all participants were given 10 min to warm up at their own pace and warmed up on their own (e.g., dynamic stretching, propulsion with and without a ball, sprinting, ball-handling, passing, and shooting). The order of execution of the tests was not specified except for the Yo-Yo 10 m recovery test, which was performed as the last of all tests. Participants were instructed to perform all tests at maximum intensity and were allowed to adequately rest (minimum of 2 min) between tests; it especially took 15 min of rest time before the Yo-Yo 10 m recovery test. The procedures for each field-based skill test were as follows. The test was explained to the players who participated for the first time, and they were allowed to practice before the test trial.

#### 2.2.1. 20 m Sprint

The participant started from a stationary position behind the starting line and, following a signal, sprinted a straight 20 m distance, as fast as possible. The sprint was performed twice within 2 min, and the best result was recorded.

#### 2.2.2. Agility T-Test (Figure 1)

We used the protocol by Yanci et al. [16], who modified the original T-turn test [17] for wheelchair users. The participants stood still, with the caster wheels behind the starting line, facing pylon A. After a signal, the participant pushed forward as quickly as possible to pylon B and touched the top of it. The player then turned left and moved to pylons C, B, and D (in order), touching the top of each pylon, and finally returned to pylon A. The time to complete the sequence was measured. The participant performed the test twice, and the best result was recorded.

#### 2.2.3. Figure-Eight with a Ball Test (Figure 2)

We used the protocol reported by Vanlandewijck et al. [18]. Following a signal, the participant moved the wheelchair around two cones in a figure-eight shape while dribbling. The participant was required to push as fast as possible, adhering to the IWBF rules for dribbling. The cones were positioned 5 m apart from each other. The time taken to complete five laps was determined. The participant performed the test twice, and the best result was recorded.

#### 2.2.4. Yo-Yo 10 m Recovery Test (Figure 3)

The Yo-Yo intermittent recovery test (level 1) was conducted according to the modified method presented by Yanci et al. [16]. As propelling speed is slower than running speed, the distance covered in the shuttle run was reduced to 10 m (it was 20 m in the original version). The participant was required to complete as many shuttle runs as possible, going back and forth between the lines in accordance with a beeping sound. The test ended when the participant failed twice to reach the goal line in time. The total distance covered during the test was recorded.

#### 2.2.5. Maximal Pass

The participant positioned the front casters behind the baseline and threw a ball as far as possible from a stationary position. We evaluated three types of passes: chest, baseball, and hook pass. The chest pass originated from the chest and used both hands. The baseball pass was a one-handed pass that used the same motion as that in the baseball throw. The hook pass was also a one-handed pass, starting from the lateral side of the player’s trunk. The participants held the ball at shoulder level with one arm extended, then lifted the arm and passed the ball over their head, followed by a throw to the target. The participants performed each of the three passes twice. The distance between the baseline and the point at which the ball hit the floor was measured.

### 2.3. Statistical Analysis

To eliminate the influence of inexperience and aging, we analyzed the player’s highest record during the four-year period for each test item. We first performed one-way analyses of variance (ANOVAs) to compare demographic and field-based test variables among the four functional classes after confirming that the data had a normal distribution (Shapiro-Wilk test) and equal variance (Levene’s test). The effect sizes in the ANOVA were displayed as η^2^, with 0.01, 0.06, and 0.14 values of η^2^ reflecting small, medium, and large effects, respectively. In cases of significance, post hoc analyses were performed using Bonferroni’s correction for multiple comparisons. The effect size was evaluated using Cohen’s d, with Cohen’s d values of 0.2, 0.5, and 0.8 reflecting small, medium, and large effects, respectively [19].

Furthermore, we performed a multiple linear regression analysis to further evaluate the relationships between the functional classification levels and field-test variables, age, years of wheelchair basketball experience, height, and arm span. Collinearity and autocorrelation were controlled using the tolerance test and Durbin-Watson test, respectively, whereas further assumptions were controlled by means of Q-Q and residual scatterplots. Data analysis was performed using the SPSS version 24.0 package (IBM Japan Inc., Tokyo, Japan).

## 3. Results

The characteristics of the participants, including the functional class, are summarized in Table 1.

**Figure 4:**
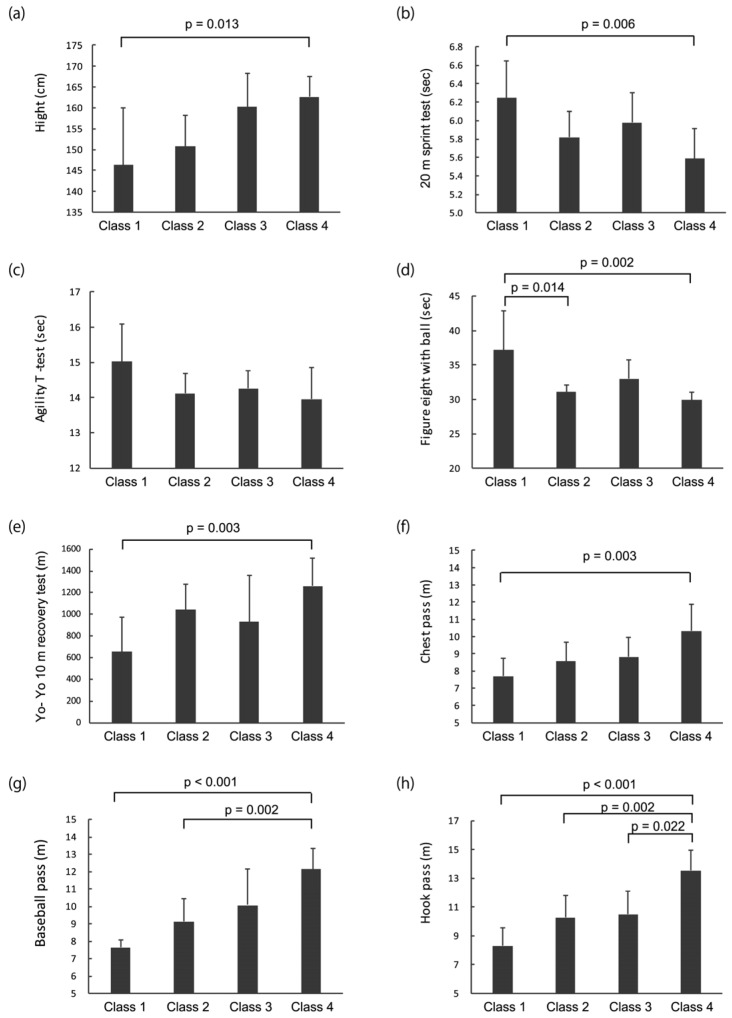
Results of the multiple-comparison post hoc analyses. (**a**) Height; (**b**) 20 m sprint; (**c**) agility T-test; (**d**) figure-eight with a ball test; (**e**) Yo-Yo 10 m recovery test; (**f**) chest pass; (**g**) baseball pass; and (**h**) hook pass.

Demographics and field-based skill test performance are shown according to the functional classification level in Table 2. Significant differences among the four functional classes were found for six test items; the agility T-test was the exception. Post hoc comparisons showed significant differences between classes for several test items (Figure 4). In particular, the figure-eight with a ball test (class 1 vs. 2, d = 1.05; class 1 vs. 4, d = 1.08), baseball pass (class 1 vs. 4, d = 3.62; class 2 vs. 4, d = 2.04), and hook pass test (class 1 vs. 4, d = 3.73; class 2 vs. 4, d = 2.09; class 3 vs. 4, d = 1.91) showed significant differences among the classes. On the other hand, for height (d = 1.6), performance in the 20 m sprint (d = 1.81), Yo-Yo 10 m recovery test (d = 1.81), and chest pass (d = 1.94), the significant difference was found only between class 1 and class 4.

The results of the multiple regression analysis are shown in Table 3. The baseball pass by the dominant hand had the largest standard partial regression coefficient (β) (β = 0.508, *p* = 0.029). The 20 m sprint, chest pass, and hook pass were excluded from the multiple regression analysis because of collinearity and autocorrelation.

## 4. Discussion

In this study, the field-based skill test variables were obviously influenced by the functional classification level; however, the degree of influence differed depending on the test item. For test items that evaluated sprint and agility, the significant difference was observed between the lowest class and highest class. However, for test of pass skills, the results showed significant differences among several classes.

We examined three types of maximal passes in this study: the chest, baseball, and hook pass, as the vast majority of passes in wheelchair basketball are caught and made using one hand to protect the ball from a defender and to throw further [20]. Although the influence of trunk stability or trunk movement may differ depending on the type of pass, previous studies have not examined the relationship between the performance of various passes and the functional classification level. However, we found that the test items with the strongest relationship to the functional classification level were the one-handed baseball pass and hook pass. Throwing the ball with one hand similarly to the baseball pass or hook pass requires considerable trunk balance in the horizontal and frontal planes. The ability to handle the ball near the lateral space of the body is a key feature that distinguishes class 3 from class 4 [20]. These results suggest that the one-handed pass is a fundamental skill, best reflecting the differences among the functional classification levels. Thus, coaches should consider the effect of the player’s functional ability in interpreting the test results for one-handed passes.

In contrast, chest pass performance significantly differed only between classes 1 and 4, consistent with previous studies that reported a difference in chest pass performance between low-point players (classes 1–2.5) and high-point players (classes 3.0–4.5) [12,15]. In the chest pass test, players throw the ball straight forward with both hands; thus, trunk rotation or lateral flexion movement is not required. Players in classes 3.0 to 4.5 are primarily differentiated by trunk stabilization in the frontal plane, and this difference is remarkable, for example, in ball rebounding situations [3]. Thus, compared to the one-handed pass test, the chest pass test is less affected by trunk stability.

We also found a moderate difference among the classes in performance on the figure-eight with a ball test, which requires dribbling skills. Molik et al. [12] compared the performance of the slalom with and without a ball among classes but found a less clear effect of the functional classification level than in the present study. The figure-eight with a ball test requires more abrupt turning than the slalom test does. To obtain a faster turn, players need to keep the trunk stable, which may have better clarified the influence of the functional classification level. Quick turning with dribbling is often used in real game situations; thus, it is helpful to observe and evaluate the player’s dribbling skills.

In this study, no significant differences were found in the agility T-test among the functional classes. Moreover, although performance in the 20 m sprint and Yo-Yo 10 m recovery test significantly differed between classes 1 and 4, there were no significant differences between adjacent classes (i.e., between classes 1 and 2, classes 2 and 3, and classes 3 and 4). These findings are consistent with those of previous studies [3,12,13,15,16]. Gil et al. [13] reported that performance in the 5 m sprint, 20 m sprint, and agility T-test is correlated with the years of wheelchair use. Additionally, Molik et al. [12] reported that performance in the 5 m sprint and shooting test did not correlate with the athlete’s classification level. These findings indicate that the abilities of speed, agility, and endurance are more influenced by experience with wheelchair use than by the functional classification level. Therefore, these items may reflect the athlete’s effort and training results.

Finally, several previous studies have reported that the player’s performance statistics during real games and current classification levels do not match the actual functional potential of the player [3,14,21,22]. However, in the functional classification system, the classifier determines the class by the volume of action from a kinematic point of view and not from the player’s game performance itself. Game statistics are considered to reflect the training outcomes for each player, the team, or role of each player in the team [23,24]. If the class is determined solely by the performance in the game and not the volume of action, players who move faster, pass further, and shoot with a higher field-goal percentage are assigned a misrepresented higher classification. Coaches and classifiers need to accurately evaluate and distinguish the effects of an individual athlete’s functional disability from the results of their efforts. It may be worthwhile to test specific skills throughout team practices to obtain a complete picture of the relationship between skill acquisition and game performance [22]. Our findings regarding the influence of functional classification level on performance in field-based skill tests provide useful information for the evaluation of individual players.

This study has some limitations. First, this study includes a small sample size, which was primarily due to the small number of female wheelchair basketball players and the goal of managing the competition level of the participants. Second, we could not take into account the sitting height of the players (i.e., the vertical grip reach from a seated position) [14]. The sitting position is strongly related to the player’s physical function, especially pelvic stability [1]. In further studies, the effect of wheelchair settings on skill performance should be considered in terms of biomechanics [6]. Finally, we employed a skill test battery derived from several works [16,18,25,26] that examined its validity and reliability. However, to better understand wheelchair basketball and its functional classification, we must continue exploring how to best evaluate sport-specific skills, especially wheelchair mobility performance [27], and measure these skills using new technologies [28,29,30].

## 5. Conclusions

We found that field-based skill test items most influenced by the functional classification levels of elite female wheelchair basketball athletes were one-handed passes (baseball and hook passes). Performance in the figure-eight with a ball test was also related to the functional classification level. When interpreting field-based test results, it is important to realize that the effect of classification levels varies from item to item because the degree of demand for trunk stability is different depending on the motion of each skill.

## Figures and Tables

**Figure 1:**
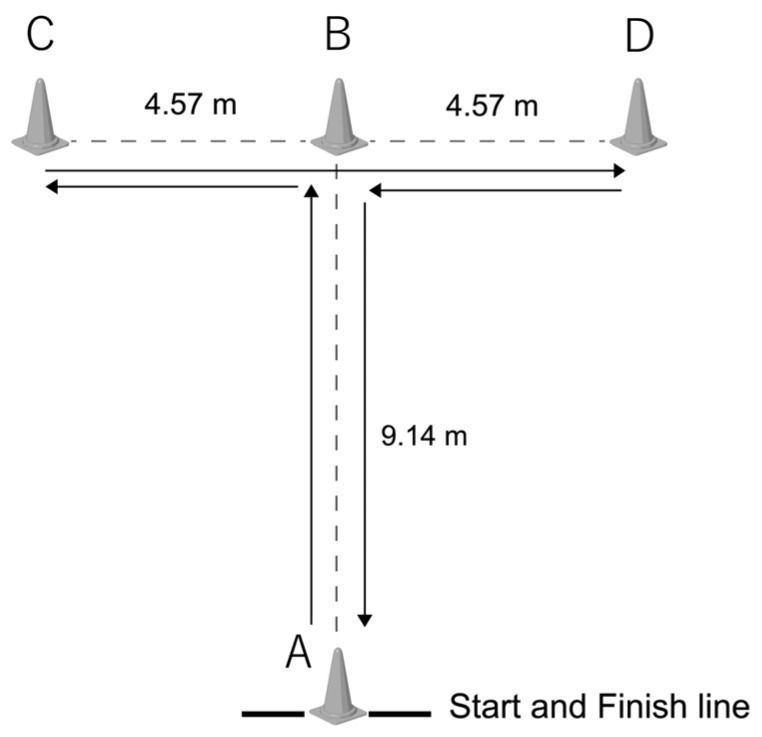
Agility T-test. After a signal, the participant pushed forward as quickly as possible to pylon B and touched the top of it. The player then turned left and moved to pylons C, B, D (in order), touching the top of each pylon, and finally returned to pylon A.

**Figure 2:**
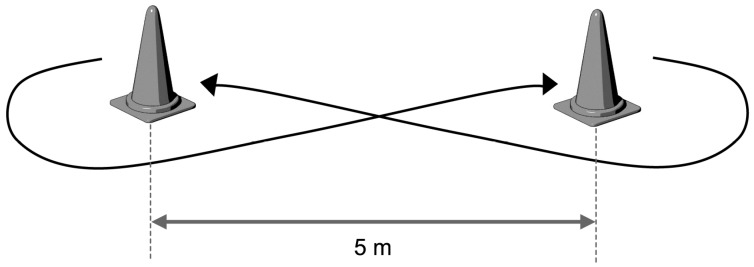
Figure-eight with a ball test. Following a signal, the participant moved the wheelchair around the two cones as fast as possible in a figure-eight shape while dribbling. The cones were positioned 5 m apart from each other. The time taken to complete five laps was recorded.

**Figure 3:**
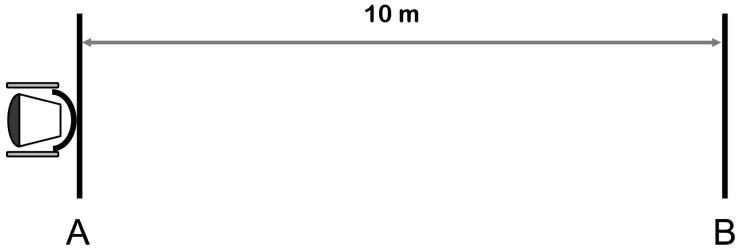
Yo-Yo 10 m recovery test. The participant was required to complete as many shuttle runs as possible, going back and forth between the lines in accordance with a beeping sound. The test ended when the participant failed twice to reach the goal line in time. The total distance covered during the test was recorded.

**Table 1:** Characteristics of the participants.

	*N*	Mean ± SD or %
age (years)	26	31.2 ± 8.0
wheelchair basketball experience (years)	26	10.7 ± 6.4
height (cm)	26	154.7 ± 11.0
arm span (cm)	26	160.8 ± 6.3
underlying disease		
spinal cord injury	12	46.2%
spina bifida	6	23.1%
skeletal system disorder	6	23.1%
amputation	2	7.7%
class (IWBF classification)		
class 1 (classes 1.0 and 1.5)	7	26.9%
class 2 (classes 2.0 and 2.5)	7	26.9%
class 3 (classes 3.0 and 3.5)	4	15.4%
class 4 (classes 4.0 and 4.5)	8	30.8%

IWBF, International Wheelchair Basketball Federation; SD, standard deviation.

**Table 2:** Demographic and field-based test data according to the functional classification.

	Class 1	Class 2	Class 3	Class 4	ANOVA		Effect Size
	(*n* = 7)	(*n* = 7)	(*n* = 4)	(*n* = 8)	F	P	η^2^
age (years)	29.7 ± 11.07	30.0 ± 7.4	37.5 ± 3.9	30.25 ± 6.6	0.992	0.415	0.12
wheelchair basketball experience (years)	12.9 ± 8.23	9.9 ± 5.0	12.5 ± 8.2	8.63 ± 5.2	0.657	0.587	0.08
height (cm)	146.35 ± 13.7	150.8 ± 7.5	160.2 ± 8.2	162.7 ± 4.9	5.469 ^	0.019 *	0.40
arm span (cm)	159.5 ± 5.5	157.5 ± 7.2	163.0 ± 4.4	163.7 ± 6.3	1.528	0.235	0.17
grip power of dominant hand (kg)	32.94 ± 5.323	33.0 ± 4.9	33.1 ± 0.9	32.9 ± 5.2	0.002	1.000	0.00
20 m sprint (s)	6.25 ± 0.40	5.82 ± 0.28	5.98 ± 0.32	5.59 ± 0.33	4.971	0.009 *	0.40
agility *t*-test (s)	15.02 ± 1.08	14.12 ± 0.56	14.26 ± 0.50	13.95 ± 0.90	2.288	0.107	0.24
figure-eight with a ball test (s)	37.15 ± 5.79	31.07 ± 1.09	33.00 ± 2.71	29.93 ± 1.14	4.647 ^	0.031 *	0.48
Yo-Yo 10 m recovery test (m)	657.14 ± 288.3	1042.9 ± 231.4	930.0 ± 429.1	1250.0 ± 240.5	5.570	0.005 *	0.43
maximal pass							
chest pass (m)	7.7 ± 1.1	8.6 ± 1.1	8.8 ± 1.2	10.3 ± 1.5	5.704	0.005 *	0.44
baseball pass by dominant hand (m)	7.6 ± 0.4	9.1 ± 1.3	10.0 ± 2.1	12.3 ± 1.7	18.720 ^	<0.001 *	0.66
hook pass by dominant hand (m)	8.3 ± 1.3	10.3 ± 1.6	10.5 ± 1.6	13.5 ± 1.4	15.721	<0.001 *	0.68

Data are presented as means ± SD. * *p* < 0.05; ^ Welch’s correction was used for *p* < 0.05 in Levene’s test; ANOVA, analysis of variance

**Table 3:** Results of multiple regression analysis examining the influencing factors of the functional classification level.

	B	SE	β	*P*	95% CI		
Constant	−2.001	5.905		0.739	−14.459	-	10.457
age	0.001	0.027	0.004	0.984	−0.056	-	0.057
wheelchair basketball experience	0.004	0.036	0.020	0.917	−0.072	-	0.080
height	0.044	0.024	0.399	0.082	−0.006	-	0.094
grip power of dominant hand	−0.005	0.054	−0.017	0.934	−0.118	-	0.109
arm span	−0.025	0.038	−0.129	0.525	−0.105	-	0.055
agility *t*-test	0.15	0.359	0.112	0.680	−0.607	-	0.908
figure-eight with ball	−0.092	0.066	−0.326	0.181	−0.231	-	0.047
shoulder pass by the dominant hand	0.268	0.112	0.508	0.029	0.031	-	0.505

R^2^ = 0.749; Durbin-Watson = 1.328; ANOVA *p* = 0.001; B: partial regression coefficient, β: standardized partial regression coefficient.
